# Indigenous Mothers’ Use of Web- and App-Based Information Sources to Support Healthy Parenting and Infant Health in Canada: Interpretive Description

**DOI:** 10.2196/16145

**Published:** 2021-05-21

**Authors:** Amy Lynn Wright, Rachel VanEvery, Vicky Miller

**Affiliations:** 1 Lawrence S Bloomberg Faculty of Nursing University of Toronto Toronto, ON Canada; 2 McMaster University Hamilton, ON Canada; 3 Hamilton Regional Indian Centre Hamilton, ON Canada

**Keywords:** Indigenous health, infant health, mothers, parenting, qualitative research, health education, health services accessibility, mobile phone

## Abstract

**Background:**

Web-based sources of health information are widely used by parents to support healthy parenting and aid in decision making about their infants’ health. Although fraught with challenges such as misinformation, if used appropriately, web-based resources can improve access to health education and promote healthy choices. How Indigenous mothers use web-based information to support their parenting and infants’ health has not yet been investigated; however, web-based modalities may be important methods for mitigating the reduced access to health care and negative health care interactions that many Indigenous people are known to experience.

**Objective:**

This study aims to understand the experience of Indigenous mothers who use web-based information to support the health of their infants.

**Methods:**

This interpretive description qualitative study used semistructured interviews and a discussion group to understand how Indigenous mothers living in Hamilton, Ontario and caring for an infant aged <2 years experienced meeting the health needs of their infants. The data presented reflect their experiences of using web-based sources of health information to support their infants’ health. The Two-Eyed Seeing approach was applied to the study design, which ensured that both western and Indigenous worldviews were considered throughout.

**Results:**

A total of 19 Indigenous mothers participated in this study. The resulting 4 themes included distrusting information, staying anonymous, using visual information to support decision making, and accessing a world of experiences. Although fewer Indigenous mothers used web-based sources of information compared to mothers in the general population in other studies, tailoring web-based modalities to meet the unique needs of Indigenous mothers is an important opportunity for supporting the health and wellness of both mothers and infants.

**Conclusions:**

Web-based information sources are commonly used among parents, and ever-evolving web-based technologies make this information increasingly available and accessible. Tailoring web-based modalities to meet the unique preferences and needs of Indigenous mothers is an important method for improving their access to reliable and accurate health care information, thereby supporting healthy parenting and promoting infant health.

## Introduction

### Background

The internet is widely used for various purposes around the world, including seeking health information. It has become so important to everyday life that some argue that access is a human right, contributing to political activism and freedom of speech [1]. In 2016, the Canadian government was the first country to recognize access to the internet as an essential service [2]. Nearly all Canadians (97%) have access to wireless internet where they live [2], and most (75%) surf the web and other web-based apps via smartphones [3]. These types of technologies make accessing the internet simple and portable, resulting in our exceedingly frequent use of web-based sources of information.

Individuals, including parents, commonly use the internet and its products to seek information pertaining to their health and to support their health-related decision making. New and expecting parents, who have grown up with access to the internet, are the most frequent users of this medium for this purpose—seeking health-related information for themselves and their infants [4]. In a study examining the use of Google, new and expecting parents were found to use this search engine more than twice as often as nonparents; they most commonly searched for health-related concerns including the health of their infants, feeding issues, and infant-related products [5]. Other studies found similar findings; new and expecting parents searched the internet most frequently for health information related to breastfeeding, infant health symptoms and how to manage them, infant development, infant nutrition, and social support [4,6-13].

However, using the internet to search for health-related information is not without challenges. In studies investigating parental use of the internet to meet their health-related informational needs, parents were commonly unable to find the information they were seeking and could not discern high-quality information from low-quality information and desired reliable web-based sources to be made available by health care providers involved in their care and the care of their children [4,7,14-16].

Indigenous mothers represent a subset of parents who are known to face unique challenges in accessing health care and health information because of experiences of racism, discrimination, and social inequity resulting from racist policies that disproportionately burden Indigenous people, particularly women [17]. Their use of search engines, web-based apps, and social media to support the health of their infants is less understood but may be an important way to promote their health and wellness by improving access to health information and promoting positive parenting and healthy choices. Few studies have investigated the utility of web-based resources for Indigenous parents. In fact, no studies have examined this phenomenon in Canada. However, research in Australia—a country that has undergone a similar period of colonization as Canada—has shown promising results, demonstrating that web-based resources can be an important way to mitigate the reduced access to health services experienced by Indigenous parents because of associated costs, wait times, and racist and discriminatory care [13,18-20]. In particular, these web-based resources are cost-effective, can reach people in rural and remote areas, are less intrusive and more confidential than traditional in-person care, and can improve communication by allowing for access in real time rather than having people wait for appointments with health care providers [18]. As is important in health initiatives with Indigenous people, health care providers and researchers can take a strengths-based approach with Indigenous mothers by acknowledging and building on existing technological skills to care for the health of their children. Furthermore, web-based apps and resources can be tailored to meet the unique needs of distinct Indigenous communities and groups, minimize language barriers, and integrate local traditional and cultural health information into accessible resources that support maternal and child health.

### Objectives

The data presented here describe how Indigenous mothers living in an urban city in Ontario, Canada use the internet, web-based apps, and social media to support the health of their infants. Indigenous people in Canada are recognized by the Constitution as First Nation, Métis, and Inuit groups, and within each group, there are numerous distinct communities with diverse cultures, languages, and traditions [21]. Although originating from a study not explicitly designed to elicit a detailed account of mothers’ experiences of using web-based information to meet the health needs of their infants, mothers nevertheless shared important insights into their needs related to web-based information sources. The results presented here aim to provide an initial understanding of this phenomenon, which has important implications for health care providers seeking to improve access to reliable health information and health promotional opportunities for these families.

## Methods

### Overview

This study used a qualitative methodology, namely, interpretive description [22], to explore the following research question: how do Indigenous mothers living in an urban city in Canada experience using health services to meet the health needs of their infants (aged ≤2 years)? Interpretive description is an applied approach to research that stems from nursing philosophy and seeks to create knowledge that can influence real-world changes for clinicians and patients [22]. This research also applied Two-Eyed Seeing, a framework that bridges both western and Indigenous worldviews to understand a phenomenon [23]. The first author is a non-Indigenous nurse practitioner of settler, European descent who strives to engage ethically with Indigenous people to further Indigenous research priorities that promote community health and well-being. The second author is a First Nations nurse and PhD student who participated in this study as a research assistant. The third author is First Nations and a manager specializing in child and family advocacy at the Hamilton Indigenous Friendship Centre. In keeping with the Two-Eyed Seeing approach, this study was collaboratively designed by both authors, a Métis scholar who participated in the lead researcher’s PhD committee, and members of the Hamilton Indigenous community. A full description of how the Two-Eyed Seeing approach was applied to this study can be found in another publication [24].

### Participant Sample

Participant mothers were recruited from local Indigenous organizations, including the local friendship center and women’s center, among others, through word of mouth and flyers posted at local health services. Mothers were included if they self-identified as Indigenous (First Nations, Métis, and Inuit), lived in Hamilton, Ontario and were caring for an infant aged <2 years. Data collection and analysis occurred simultaneously, and recruitment continued until conceptual redundancy was achieved and data were sufficiently detailed to answer the research question [25].

### Data Collection

Data were collected through semistructured interviews, which lasted approximately 60-90 minutes with each participant mother and the first author at a location of convenience for the mother. All mothers were offered the presence of a research assistant, but only one mother requested this. All mothers spoke English as their first language. Interview questions were developed using *Behavioral Model and Access to Medical Care* [26] by Andersen to ensure that questions incorporated the wide breadth of factors described in the model and known to influence an individual’s access to health care, including environmental factors, characteristics of the population, health-related behaviors, and the health status of the individual. Andersen model has been widely tested and validated with Indigenous and other ethnic groups [26]. Some examples of interview questions are as follows: (1) “What do you do to help your baby stay healthy; (2) “What services or sources of information do you find helpful in meeting the health needs of your baby; and (3) “Tell me more about how you use [app or website] to address your baby’s health needs?” Once all interviews were completed, participating mothers were asked to come together as a group to review the initial study findings and further clarify evolving concepts. All mothers were invited, and 8 participated. Mothers verified the initial study findings and did not ask for any data to be removed. Written and verbal consent for study participation was collected when they first met each mother. Interviews were audio recorded, transcribed, and then uploaded onto NVivo 12 (QSR International) for qualitative data analysis [27]. Field notes were collected during and after each interview and during and after the discussion group. These provided context, including mothers’ emotional reactions during the interviews, and added depth and meaning to the transcripts. Data were stored in a locked filing cabinet and a password-protected computer in a locked office at the university. Ethics board approval was obtained from 3 local research ethics boards, including the Hamilton Integrated Research Ethics Board, Mohawk College Ethics Board, and the McMaster University Family Medicine Program, and the staff at the local Friendship center collaborated on and approved the study design.

### Data Analysis

An iterative thematic analysis of the data was conducted by the lead researcher. Transcripts were read and reread and then coded according to the techniques described by Saldana [28], which allowed for pattern recognition and exploration of concepts, relationships, and meanings. The results presented here constitute a thematic summary of the data pertaining to mothers’ use of web-based resources, including the internet, apps, and social media sources, and has been presented in a way that both provides an understanding of the phenomenon and supports researchers and clinicians applying these findings to real-world applications to meet the health information needs of Indigenous mothers and their infants.

## Results

### Overview

The understanding of how Indigenous mothers in Hamilton, Ontario use web-based resources to meet the health needs of their infants is informed by interviews with 19 mothers. The demographic details are presented in Table 1. The mothers’ number of children and education levels have been compared with local data for First Nations people [29], and the proportions of First Nations, Métis, and Inuit identities have been compared with the identities of self-identifying Indigenous people living in Hamilton [30].

Briefly, 15 mothers were identified as First Nations, 2 as Métis, and 2 were unsure of their specific Indigenous heritage, as is not uncommon for Indigenous people, due to the lasting impacts of colonization, forced assimilation, and resulting loss of identity and cultural ties. The mean age of participants was 28 years (SD 5.82 years), and nearly one-third were first-time mothers. When asked whether they used the internet or other web-based sources to answer health-related questions regarding their infants, mothers reported solely using Google as their internet search engine and Facebook as their primary social media platform. No other search engines or social media platforms were mentioned by the mothers. Within the group of 19 mothers, 10 (53%) reported using Google, 4 (21%) reported using a health-related app, 5 (26%) used Facebook, and 6 (32%) did not use the internet in any form to address their informational needs regarding their infants’ health. Of the 6 (32%) mothers who used multiple internet sources, 2 (11%) used both Google and Facebook and 4 (21%) used both Google and apps, but none used all 3 types of internet sources (Google, apps, and Facebook).

When asked about the reasons for using various types of web-based resources, mothers reported using Google to search for infant norms (developmental milestones, normal temperature, normal behavior, and sleep patterns), how to care for common illnesses (especially rashes and fevers), signs and symptoms of infections, when to take the infant to see a doctor, and baby food recipes. Mothers reported using Facebook to peruse mom-to-mom blogs, share issues with other peers, and find examples of similar experiences related to infant health. Parenting apps were used to discover infant developmental milestones, especially weekly updates on developmental changes and stages, to answer questions about normal infant health issues, guidelines for when to start feeding solids, meal planning strategies for infants, and baby food recipes.

Data analysis of mothers’ experiences of using web-based resources to meet their informational needs pertaining to their infants’ health resulted in 4 main themes: (1) distrusting information, (2) staying anonymous, (3) using visual information to support decision making, and (4) accessing a world of experiences. The following section describes these themes in more detail. Supporting quotations from these themes can be found in Textbox 1.

**Table 1:** Demographic information: participant mothers.

Characteristic			Participants (n=19), n (%)		Comparison with First Nations data in Hamilton, n (%)^a^
**Age (years)**					
	<25 years	5 (26.3)		—^b^	
	26-30 years	8 (42.1)		—	
	>31 years	6 (31.6)		—	
**Number of children^c^**					
	0	0 (0)		200 (36)	
	1-2	12 (63.2)		200 (36)	
	3	2 (10.5)		83 (15)	
	≥4	5 (26.3)		72 (13)	
**Education^c^**					
	Less than high school	9 (47.4)		316 (57)	
	Completed only high school	3 (15.8)		111 (20)	
	Some college or university	7 (36.8)		128 (23)	
**Indigenous identity^d^**					
	First Nations	15 (78.9)		9695 (67.1)	
	Métis	2 (10.5)		3960 (27.4)	
	Inuit	0 (0)		165 (1.1)	
	Unknown (not sure if First Nations, Métis, or Inuit)	2 (10.5)		—	
**Regular health care provider**					
	Family physician	17 (89.5)		—	
	Pediatrician	1 (5.3)		—	
	None	1 (5.3)		—	

^a^Number of children (% of family).

^b^Demographic data are not available for Indigenous people within the city of Hamilton.

^c^n=555; data obtained from Smylie et al [29].

^d^n=13,820; data obtained from Statistics Canada [30].

**Themes and supporting participant quotes.**

**Distrusting information**

- “Well I use the internet when he’s sick...search up his symptoms and just see. I try not to do that too much because sometimes some crazy things pop up and then I get scared.” [First Nations mother of two, aged 26 years]
- “I look up on the Internet and some of the stuff that comes up, it’s like, I didn’t think you were allowed to use that kind of stuff on kids! And I’m like, no, I would rather go to the doctors and get her looked at. Sometimes when you read stuff on the internet, it is not always accurate.” [First Nations mother of five, aged 27 years]
- “Every time you Google something it is always going to show up cancer.” [First Nations mother of two, aged 21 years]
- “Well obviously, I question them. I know I shouldn’t be on doctor Google because obviously there is conflicting information out there.” [First Nations mother of two, aged 35 years]
- “If you were to type in something like an ear infection, they will give you so many different options. Some of them say the same thing and some will say other things. Like I don’t know which one is going to be better for her or I don’t know how bad it is. I don’t always trust it.” [First Nations mother of three, aged 32 years]

**Staying anonymous**

- “They do have like mom groups [on Facebook]. I don’t ask for other people’s opinions. They ask questions...like when something is wrong with their kid or something, they’ll say ‘is this normal’? and then other moms give their opinions and then I just go over it. I don’t really talk.” [First Nations mother of four, aged 21 years]
- “Like mom-to-mom groups. I read. I don’t want to post in it because then all of a sudden you’re the center of the post and they never stop commenting. It will be a year later and someone is still commenting.” [First Nations mother of two, aged 21 years]

**Using visual information to support decision making**

- “Some moms will ask about their child and post a picture, and say if it is like a rash or say if they got chicken pox and they don’t know what it is. They will be like what is this? They post on there and other people will comment on what they think it is. But I thought [my son’s rash] was actually just a rash that he developed because he was sick. Then it was actually hives which when we went to the doctors she told me it was hives. An allergic reaction to something.” [First Nations mother of two, aged 21 years]
- “Like they will post pictures of a rash on their kid, and be like, ‘I have a doctor’s appointment later but I am just looking for advice like if anyone else’s child has ever had this?’ What do you think it looks like? Things like that.” [First Nations mother of two, aged 26 years]

**Accessing a world of experiences**

- “I kind of just read Mom blogs. They make me feel a little bit better that there are other moms out there.” [First Nations mother of two, aged 35 years]
- “Say your son has a rash, and you’re like, ‘There is this purplish-red rash, how do I get rid of it or what do I do?’ And [you get] fifteen answers from every mom who seems to be going through the exact same thing as you.” [Métis mother of one, aged 22 years]
- “What kind of laundry detergent is safest for my son’s skin? There is someone else out there in the world, whose son has the same skin as your baby or goes through the same thing as your baby.” [Métis mother of one, aged 22 years]
- “You are looking at ten different answers and there is one answer that appeals to you. Especially with your type of parenting style. There is someone who parents their children similar to you, right?” [Métis mother of one, aged 22 years]

### “Crazy Things Pop Up”: Distrusting Information

Although most mothers used web-based sources of information to answer questions they had related to their infant’s health, development, and well-being, they also struggled to trust the information they found on the web. Mothers shared the impressions that the information was often inaccurate and did not align with other health information they had received from other, more trusted sources such as health care providers. A common complaint about using Google was that symptoms of illness were commonly associated with symptoms of cancer, which scared mothers and made them anxious. Mothers shared their struggles with navigating large amounts of information and the ways they had developed to assist them in choosing the information that they could use to make decisions. Some mothers reported reading numerous answers and choosing to believe or act on the advice that they found to be most frequent and consistent with their existing knowledge of the issue. Another mother went with her *gut instinct* and chose the answers that she believed were best and were the easiest to apply or use. Other mothers shared that they chose to act on information that was in line with their own parenting beliefs and styles. Understanding that discerning quality and reputable web-based information was difficult, mothers had developed these tactics to choose the information that they could use or otherwise sought information from health care providers directly.

### “All of a Sudden You’re the Center of the Post”: Staying Anonymous

Mothers required the ability to use web-based resources with complete anonymity. Even those who used Facebook to access parenting discussion forums chose not to engage with other mothers by replying to posts or posting their own. Instead, they chose only to read other mothers’ posts and apply relevant content to their own decision making and parenting experiences. One mother expressed her frustration at being constantly notified of new posts to her thread if she initiated a discussion in a mom-to-mom forum. Rather than having these notifications persist, she chose not to post at all.

### “What Do You Think It Looks Like?” Using Visual Information to Support Decision Making

The unique ability to share and view pictures was one of the most common reasons for mothers to use web-based resources, particularly apps and Facebook discussion forums. Although Google was used to look up pictures of specific symptoms or known illnesses, apps provided pictures of developmental stages and common newborn ailments or caregiving techniques, such as providing an infant with medicine. The use of pictures assisted mothers in interpreting health information that may have otherwise been complex and technical.

Facebook discussion forums were particularly important as they allowed mothers to post pictures of their infants’ symptoms and to seek advice from thousands of other mothers worldwide. The most frequent symptom that participant mothers spoke about was their infants’ rashes. These common newborn ailments were often confusing to mothers who wanted to understand the cause and source of the rashes and whether their infants’ rashes warranted seeing a physician. The participants used these forums to find pictures of symptoms that were similar to those of their own infants, as they did not post their own pictures. With hundreds or thousands of mothers subscribing to each forum, mothers did not find it difficult to find pictures and descriptions similar to the issues they and their infants were experiencing. However, this type of self-diagnosis is problematic. One mother shared her experience of using such a forum to determine that her infant had a rash associated with an illness. However, when she visited her physician, she was surprised to discover that the rash had been caused by an allergic reaction. These types of self-diagnosis errors can be dangerous for infants if mothers do not seek advice from a health provider, instead choosing to assume that their understanding of web-based resources is correct.

### “There Is Someone Out There Going Through the Same Thing”: Accessing a World of Experiences

Finally, web-based resources, particularly Facebook discussion forums, provided mothers with access to thousands of mothers from across the world. Participant mothers shared that they had no difficulties finding mothers with similar questions, experiences, or dilemmas. This helped to normalize their experiences, boost their parenting confidence, and lessen their stress and anxiety by reassuring them that they and their infants were like others, even if those in their immediate social network were dissimilar. Although the participant mothers did not interact with other mothers on discussion forums, these forums formed a sort of web- and app-based social network, where mothers found a place in which they felt a sense of belonging and similarity with other mothers and where they felt secure in their ability to control their information and interactions through the anonymity provided by the sites.

In summary, the use of web-based sources of information for parenting and newborn health was common among participant mothers. These sources of information were not always reliable, and mothers struggled to determine which information to trust and act on among a sea of suggestions and advice from websites, apps, and Facebook discussion forums. The ability to anonymously peruse information was extremely important, as it facilitated mothers controlling their interactions and information-sharing with others. The use of pictures and other visual information assisted mothers in interpreting otherwise complex health information and also enabled seeking advice from other mothers. Finally, access to thousands of mothers across the world via Facebook discussion forums helped to normalize an otherwise isolating experience of parenting a newborn, linking mothers to others with similar experiences and challenges. Web-based resources are highly used sources of information for mothers and other new parents, and the data described here present some ways in which these sources may be tailored to meet the needs of Indigenous mothers while also mitigating the risks associated with unreliable information and self-diagnosis.

## Discussion

### Principal Findings

To the best of our knowledge, this study is the first to discuss Indigenous mothers’ use of web-based resources to support the health of their infants. This understanding is important for health care providers who wish to assist Indigenous mothers in meeting their health-related informational needs. This study and the literature has demonstrated that new and expecting parents are most commonly using internet sources of information to address questions and concerns related to parenting and newborn health, yet many are challenged by the rampant availability of misinformation, their inability to discern high-quality scores from low-quality sources, and understanding complex medical information [2,5,14,31]. Certainly, this presents an opportunity for the development of innovative internet resources to meet the needs of parents while providing reliable health information in an accessible format. In today’s health care environment, access to quality information that is easily understood by mothers, which results in safe and positive parenting choices, is essential to promoting healthy newborn development and facilitating the appropriate use of health care services. The results of this study suggest that a mother’s ability to remain anonymous while searching and interacting with web-based information is imperative, as is a visual format and access to a wide range of parenting and newborn experiences so that mothers can easily find their place of belonging among countless possibilities. The creation of web-based modalities that meet these requirements could be a valuable asset for health care providers looking to link parents with reliable health information, especially those who are vulnerable or marginalized and at a higher risk of experiencing adverse health outcomes. In addition, web-based modalities can assist health care providers by making health education more efficient and accessible.

Despite the high prevalence of access to smartphones and internet use among Canadians, our study found that just under 70% (13/19, 68%) of mothers used the internet in some form to answer health-related questions pertaining to their newborns. This number is significantly lower than many other studies that show that ≥90% of parents used the internet for this purpose [4,7,12,16]. However, our sample was small and did not reflect Indigenous people across Canada or their diverse experiences. Many mothers in this study experienced lower financial means, which may have led to text-only plans on their mobile phones and limited their opportunities to use the internet. In fact, several mothers shared that they chose not to use paid apps. Other mothers shared a preference for face-to-face interactions with health care providers, choosing not to use web-based sources because of distrust of their accuracy. Despite this, the majority were using internet sources of information; thus, web-based modalities are likely an excellent way to reach these mothers with important health education related to parenting and infant health.

The distrust of web-based information found in this study is a common finding among parents using the internet for infant-related health information [6,7,9,14,15]. Not being able to locate the desired information can have significant implications on decision making. As demonstrated in a study of mothers using the internet for breastfeeding support, mothers who were unable to find the information they were seeking were more likely to choose to feed their infant formula rather than breastfeed [14]. The mothers in this study developed tactics to evaluate the information they found on the internet, most often choosing information that was repeated on various websites or by numerous mothers on discussion forums, going with their *gut instinct* or choosing information that was agreeable to them. Other studies have found that parents used similar approaches [8,32]. However, other parents did not attempt to discriminate between correct and incorrect information at all. One study found that 74% of mothers using a pregnancy app and 68% of mothers using a parenting app chose not to validate information [33]. This lack of health literacy and critical thinking can be problematic, and similar to the mothers in this study, those with lower levels of education have been found to be less critical about the information they discover on the internet [32]. Together, these findings suggest that Indigenous mothers, similar to most parents, lack the web-based health literacy skills necessary to discern between reliable and unreliable sources of information and that health care providers and educators have an important role in teaching web-based health literacy skills to parents and providing reliable sources of information to parents that can be used appropriately to answer their parenting questions [32,33].

This study found that Indigenous mothers most commonly used Google to meet their information needs on parenting and infant health issues. Fewer mothers reported using parenting apps and Facebook. This trend is similar to other studies, in which only half of internet users used parenting apps and Facebook was the most frequently used social media platform for parents [4,33,34]. In this study, the appeal of web-based sources of information for Indigenous mothers included the availability of visual information, a web- and app-based social network, and anonymity. Similarly, Skranes et al [9] found that parents appreciated the visual nature of web-based information and most commonly searched for pictures of infant rashes. This common infant ailment presents a conundrum to parents, and accurate sources of visual information pertaining to rashes would likely be a helpful and welcome resource for them. In addition, the availability of visual information likely also supports health literacy, as parents can depend less on written information. Visual information may also be a faster and more efficient way for parents to process health-related information compared with reading.

Next, Facebook discussion forums were important to the Indigenous mothers in this study as they created a type of web- and app-based social network by instilling a sense of normalcy among thousands of other mothers with various experiences. This was particularly important for some mothers who had purposefully isolated themselves from friends and family who had negative influences in the past. Many participants had experienced negative health care encounters, racism, and discrimination within mainstream services and felt that their parenting was surveilled by child protection services. Indeed, both a lack of culturally safe health care services and Indigenous health care providers are barriers to safe and appropriate care for Indigenous families in Canada [13,17]. In the absence of culturally safe health care options, web-based discussion forums represented a safe place where they were in control of their personal information and could choose whether to anonymously view information or interact with other mothers on the forum. This phenomenon has been documented elsewhere, and other studies have found that a web- and app-based social network can provide parents with a sense of normalcy, social support, increased parenting confidence, and reduced isolation, all of which are particularly important in the postpartum period [6,8,10,35]. In a study of mothers’ use of social media, Duggan et al [34] found that only 36% of mothers asked questions, whereas others chose to anonymously view content. Discussion forums appear to serve multiple purposes for Indigenous mothers, representing a web- and app-based social network, assisting mothers in finding others with similar experiences, even when rare, and promoting a safe place where mothers can choose whether to interact or view content anonymously.

Mothers did not discuss the threat of cyberbullying as a reason why they chose not to interact with mothers on discussion forums and were not directly asked about this by the interviewer. However, in their personal lives, several mothers isolated themselves from friends and family members who they felt had a negative influence on their lives. The threat of negative interactions and the potential for cyberbullying if engaging with other mothers in web-based forums, therefore, may explain their insistence on anonymity.

Although these findings require verification with a larger sample size, they have several notable implications. First, Indigenous mothers, similar to other parents, require reliable sources of parenting and infant health information. Health care providers should suggest reputable websites to parents to assist them in making informed decisions based on high-quality information [7,32,33]. Culturally safe web-based resources and those created from an Indigenous worldview in collaboration with Indigenous communities are also imperative to best meet the needs of Indigenous families. Web-based modalities represent an important opportunity to provide health education and promote health. Second, web-based modalities such as apps should be available free of charge. Mothers and infants experiencing poverty are at high risk of experiencing adverse health outcomes; thus, their access to reliable health promotional information is paramount and should be prioritized. Charging fees to access information is prohibitive and deters mothers from using them fully, as demonstrated in this study. Third, there appears to be a missed opportunity for health care providers to monitor web-based discussion forums to ensure that accurate information is being shared and to provide reliable advice to mothers and other parents. This type of intervention would significantly improve the credibility of discussion forums, thereby amplifying the benefits of this highly used source of information.

### Future Research

Given the historical, political, and social contexts that the participant mothers experience in their daily lives, these findings are likely to be relevant to other Indigenous parents who are similarly impacted by racism, discrimination, and social inequity stemming from colonization and subsequent discriminatory policies and stereotyping. Future research is needed to validate these findings with a larger and more representative group of Indigenous parents. Conversely, recognizing the vast diversity of Indigenous people in Canada, researchers seeking to create web-based information tools and technologies should collaborate with local Indigenous communities to ensure technology and content is relevant to local cultures, languages, and traditions.

How Indigenous mothers use web-based resources for their infants’ health has not been previously described in Canada, and further research is needed to better understand parents’ preferences and challenges and how best to address these needs. As this study focused on mothers as the primary caregivers of infants, future work to address the information needs of fathers and other parents (two spirit, lesbian, gay, bisexual, transgender, queer, questioning, intersex, and asexual) is necessary. Finally, a more in-depth exploration of mothers’ hesitancy to interact on discussion forums might reveal the dangers of cyberbullying and suggest ways to mitigate these risks and promote the benefits of social interaction for otherwise isolated mothers.

### Conclusions

Parents represent one of the most frequent users of web-based information, such as Google, apps, and Facebook. This study provided initial insights into how Indigenous mothers used web-based sources of information to meet the health needs of their infants and found that web-based sources were commonly used and were helpful to mothers. Significant challenges existed for mothers who navigated vast amounts of web-based information and attempted to interpret and apply the information to their lives. Health care providers should provide parents with reputable sources of web-based information pertaining to healthy parenting and infant health to assist parents in addressing their questions and making decisions about their infants’ health. Web-based modalities of health information appear to be useful opportunities to provide health education and health promotion to Indigenous mothers, and researchers and health care providers seeking to create these types of technologies should collaborate with local Indigenous communities to ensure relevancy.
